# Spider Diversity on the Oceanic Island of Fernando De Noronha, Brazil, and Implications for Species Conservation

**DOI:** 10.1673/031.013.14801

**Published:** 2013-12-11

**Authors:** Gilson Carlos da Conceição Freitas, Antonio Domingos Brescovit, Simao Dias Vasconceloslc

**Affiliations:** 1 Departamento de Zoología, Universidade Federal de Pernambuco. Recife, PE, Brazil; 2 Laboratório Especial de Coleções Zoológicas, Instituto Butantan. Sao Paulo, SP, Brazil

**Keywords:** Araneae, arthropod biodiversity, guild, invasive species, island biota

## Abstract

Fernando de Noronha is an oceanic archipelago in Brazil that has been subjected to major alterations in its natural habitat, as it is exposed to increasing rates of tourism. This research aimed at performing the first survey of spider species on the main island, focusing on the environmental occupation and conservation status of local species. Spiders were sampled through pitfall traps, beating sheets, and active collection in the dry (October 2005) and rainy (April 2006) seasons in several parts of the island, such as urban and protected areas. A total of 1,532 adult spiders from 44 species distributed in 20 families were collected. Forty-two species are newly recorded on the archipelago, of which 10 appear to be native. Theridiidae and Salticidae were the richest families, with seven and five species respectively. *Hogna* sp., probably an endemic Lycosidae species, had the highest abundance throughout the study (17%). Several non-native species were found, especially in the surroundings of human habitations. Areas exposed to human settlements had higher diversity indices and evenness values when compared to preserved areas. Most species were classified as being diurnal space web-weavers. The results suggest that non-native species seemed to be established on the island, due mainly to the traffic of people and goods from the continent.

## Introduction

Islands have been treated as “model systems” to provide insights into ecological and evolutionary processes, including temporal patterns of extinction (Triantis et al. 2010). Insular systems often contain unique species assemblages and frequently harbor remnant populations that have been eliminated from surrounding mainland areas (Drake et al. 2002). In Darwinian islands, that is, oceanic islands that arose from the floor of the ocean basin and were never connected to continental landmasses, colonization of species increases at a rate that depends on time and the interplay of spatial isolation (Gillespie and Roderick 2002). In their natural state, oceanic islands support a considerable proportion of endemic species, many of which may be experiencing dramatic threats as a direct consequence of human habitation (Paulay 1994; Martin et al. 2010; Triantis et al. 2010), especially since distant islands are known for their large numbers of endangered species and high rates of extinction (Gillespie et al. 2008).

The Brazilian oceanic archipelago of Fernando de Noronha is classified as a World Natural Heritage site by UNESCO, as the islands play a key role in the process of reproduction, dispersal, and colonization by marine organisms in the Tropical South Atlantic (UNESCO 2007). Officially discovered by Portuguese navigators in 1503, it has also been exploited by European and Brazilian colonizers with irreversible destruction of native vegetation. Since its first permanent human settlements in the 16^th^ century, it has suffered major ecological perturbations from a number of invasive animal and plant species that have been introduced to the islands (Teixeira et al. 2003). Tourism is now a major economic activity in the islands.

Although officially protected by Brazilian environmental law, the archipelago faces challenges to minimize anthropogenic impacts on its biota, prompting the need for inventories on its fauna. So far, no systematic survey on invertebrate diversity has been performed, leaving unanswered questions about ecological processes of invasion, establishment, and extinction of key taxa such as arachnids.

Spiders are key predators in terrestrial ecosystems and are a megadiverse group with more than 40,000 described species, occurring in all continents except Antarctica (Platnick 2013). Araneae are sensitive to environmental alteration and, as generalist predators, influence herbivore and detritivore populations, so their abundance and richness can reflect those of taxonomic groups belonging to lower trophic levels (New 1999). Moreover, spiders can explore a myriad of environments, occupy a key position in a variety of food webs, and are ubiquitous and relatively easy to collect and identify to morphospecies (Oliver and Beattie 1993).

The concept of guild involves grouping organisms that significantly overlap their niche requirements and exploit the same environmental resources (Root 1967). Spiders can be classified into guilds based on family-level determination, which reflects their foraging manner, web type, microhabitat use, and activity patterns (Uetz et al. 1999). Guild classification is a useful tool in ecological studies that seek to describe diversity in communities and functional relationships.

The diversity of species in oceanic islands is related to island size and degree of isolation, larger islands being associated with more species and reduced extinction rates (Paulay 1994; Gillespie 2002). The small size of Fernando de Noronha, associated with the process of anthropogenic disturbance, has led to the hypothesis that areas protected from human presence will support a small, endemic spider fauna, when compared to areas exposed to human-mediated processes, which may support a richer fauna, composed of species widely distributed.

In this context, this research aimed to: i) perform the first inventory of spider species in the oceanic island of Fernando de Noronha, an increasingly endangered hotspot for biodiversity conservation; ii) detect the presence of non-native species; and iii) compare, in a short-term scale, spider communities in areas with different degrees of human presence.

## Materials and Methods

### Description of the area

**Figure 1.**
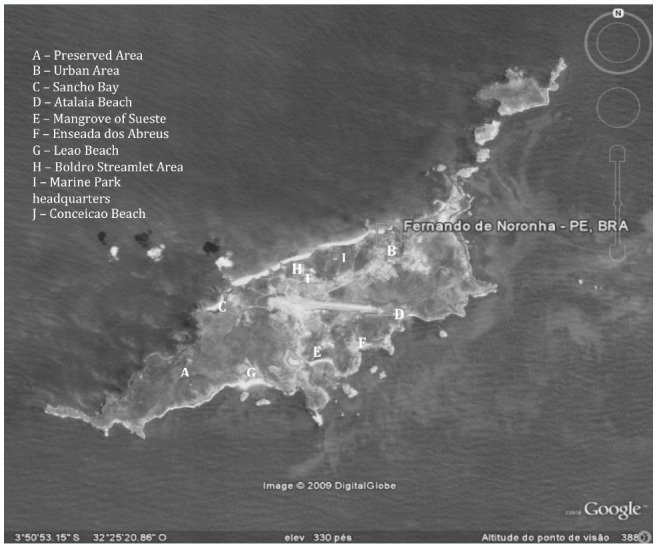
Distribution of collection points on Fernando de Noronha Island. High quality figures are available online.

The archipelago of Fernando de Noronha lies just off the rim of the continental shelf (3° 50′ S; 32° 15′ W), 345 km northeast of the Brazilian mainland (Figure 1). It is composed of 21 islands and islets, of which the only inhabited one (also called Fernando de Noronha) has a population of ca. 3,200. The total land area is 18.4 km^2^, of which 16.9 km^2^ belong to the main island (Teixeira et al. 2003). The whole archipelago is legally protected and comprises three areas of environmental preservation in which human activities (subsistence agriculture, commerce, and tourism) are limited or prohibited.

The climate is tropical, with two well-defined seasons, dry (August to January, mean precipitation 27.2 mm/month) and rainy (February to July, mean precipitation 211.7 mm/ month). The temperature ranges from 23.5° C to 31.5° C, with an annual mean of 27.0° C (Ibama 2006). The soil is shallow and provides little water retention. Native flora, characterized as seasonal deciduous vegetation, is sparse and primarily represented by bushes and herbs, with several introduced plant species (Teixeira et al. 2003). Overall, the island suffers from a shortage of water, as there are no permanent freshwater reservoirs.

Ten sites on the island of Fernando de Noronha were sampled in two expeditions that encompassed the highest (April) and lowest (October) rainfall periods. Each expedition lasted 15 days. The sites were selected based on the following criteria: accessibility, diversity of vegetation and soil characteristics, degree of exposure to human activities, and geographical position on the island. To draw inferences on the relationship between the diversity of communities and anthropogenic activities, the sampling covered virtually all types of habitat on the island (Figure 1). Human impact on collecting sites was categorized as low, intermediate, or high, based on the frequency of area usage, access (since part of the island is open only to scientific and management activities), presence of buildings, economic activities, and number of tourists.

Sites under low anthropogenic impact include (letters in parenthesis refer to Figure 1): Preserved area (A): the most restricted access area in the archipelago, located in National Marine Park; vegetation has a secondary stage of succession with predominance of climbing species *Ipomoea nil* (L.) Roth (Solanales: Convolvulaceae), *I. hederifolia* L., and *Cissus verticilata* (L.) (Vitales: Vitaceae)

*Sancho Bay* (C): a little bay situated in an area of difficult access, exposed to limited tourism; sparse vegetation, with predominance of the endemic *Cereas insularis* Hemsl (Caryophyllales: Cactaceae).

*Atalaia Beach* (D): part of National Marine Park, it is a restricted area in which access to the beach is open only for 2 hr/day for small groups of visitors; *C. insularis* is also present.

*Mangrove of Sueste* (E): a unique mangrove in the Southern Atlantic islands, its vegetation is composed almost exclusively of *Laguncularia racemosa* (L.) (Myrtales: Combretaceae); limited human presence, although some garbage was found in the area.

*Abreus Cove* (F): situated between Atalaia Beach and Sueste Bay, this rocky area has herbaceous vegetation, with low grass cover and presence of *C. insularis;* it is a nesting site for several bird species, especially *Sula sula* (L.) (Suliformes: Sulidae).

*Boldró Streamlet Area* (H): an area surrounding a streamlet formed by the drained water that runs down the higher parts of the island; riparian vegetation is composed of centuries-old trees, namely *Erythrina aurantiaca* Ridl. (Fabales: Fabaceae) (Figure 1).

Sites under moderate anthropogenic impact included:

*Leão Beach* (G): the most important site for sea turtle oviposition on the island, it is also a site for tourism and diving, and there is a small lodge for visiting scientists; vegetation in this beach and its surroundings is composed of low shrubs and sparsely distributed trees.

*Conceição Beach* (J): the largest and most easily accessed beach in the island, it is intensively used by tourists; vegetation is typical of the *Restinga,* an ecosystem of the Atlantic rainforest biome composed mainly of pioneer formations tolerant to coastal conditions; shrubs dominate the area, with a few trees on its boundary.

Two sites were classified as under high human impact:

*Urban Area* (B): the village where ca. 70% of the population of the island lives, comprised of housing, roads, and the airport; litter accumulation can be found in several areas.

*National Marine Park Headquarters* (I): this comprises the administrative headquarters of National Marine Park, including the accommodation for employees and visiting scientists, an electricity generation station, and a water purification/distribution plant. Despite the human presence, it is not considered to be an “urban” area given the paucity of permanent residents; several plant species, mainly ornamental shrubs, have been introduced in the area.

### Collection and identification of spiders

To maximize the likelihood of collecting individuals from different plant strata and with different foraging behaviors, three methods were used: *i)* pitfall traps, *ii)* beating sheets, and *iii)* active search for diurnal and nocturnal species (Coddington et al. 1991). Pitfall traps consisted of 500 mL plastic cups containing 200 mL of 70% alcohol (7 cm diameter) buried in the ground at rim level and were scattered throughout the island, disposed to maximize capture. A total of 324 pitfall traps were scattered throughout the 10 sampling sites in the island, with a minimum distance of 5 m between adjacent traps. Pitfall traps were left in the field for 72 hr and each collection corresponded to a sample. A 1 m^2^ white beating sheet was used to capture specimens from shrubs and trees; each sample consisted of 20 minutes of collection between 08:00 and 12:00. Given the dominantly low vegetation in the island, sampling focused on branches up to 2.5 m high. Active collection took place within a 200 m radius from the pitfall trap sites.

Active searching involved manual collection using soft forceps and focused on specimens found on the soil and on vegetation up to 2 m high. For nocturnal manual collection, the area enclosed within a 30 m long and 10 m wide rectangle was sampled for a 1 hr period between 20:00 and 23:00. Diurnal collection consisted of searching soil and vegetation for a 1 hr period (09:00–10:00) per sample, collecting every spider found.

All sampling methods were performed in both seasons. Since it was not possible to sample all areas equally (for logistical reasons) or more intensely (due to the restrictions imposed by the preserved status of the island), two areas with the most dissimilar degrees of anthropogenic pressure, the preserved and the urban area, were chosen for a direct comparison of ground living spider communities. Both areas were exposed to the same collecting effort by deploying pitfall traps in a 225 m^2^ area at each site.

Specimens were identified by the authors and deposited in the arachnid collection of the Instituto Butantan, São Paulo (curator: D.M. Barros Battesti).

### Data analysis

Species accumulation curves were plotted against sampling efforts for passive (pitfall traps) and active (beating sheet, active search) collecting methods, as well as for the data rep-resenting a combination of both methods. Non-parametric estimators Chao 1, Chao 2, Jacknife 1, and Jacknife 2 were calculated to predict the number of species based on the numbers of singletons, doubletons, and the number of times an individual of each specimen was captured (Chao 1984; Colwell and Coddington 1994). Also, Shannon's diversity index (H') was calculated. Statistical analyses were performed using ANOVA and the Mann-Whitney test to evaluate differences in the richness and abundance between areas (α = 0.05). Only the numbers of adult spiders were included in the statistical analyses, since young individuals cannot be identified at species level.

Spider species were categorized in guilds following Dias et al. (2010), who established the most common guilds in three major Neotropical ecosystems. This categorization is based on web use (presence/absence of web foraging behavior), type of web (sheet, spatial, orb), foraging manner (ambusher, stalker, active runner), micro-habitat use (ground, vegetation), and diel activity (diurnal, nocturnal).

## Results

A total of 3,369 spiders belonging to 44 species and 20 families were collected, of which 1,532 (45.5%) were adults and 1,837 (54.5%) were juveniles. The richest families were Theridiidae (7 spp.), Salticidae (5 spp.), Araneidae (4 spp.), Oonopidae (4 spp.), and Pholcidae (4 spp.), which combined made up 54% of the total species. In terms of abundance, three families, Oonopidae, Theridiidae, and Lycosidae, combined represented 55.9% of total adult specimens (Table 1). Oonopidae was the most abundant family, comprising 23% of all adults collected, and third in richness, along with Pholcidae and Araneidae (9% of total species each). Three families, Corinnidae, Miturgidae, and Theraphosidae, were represented only by juveniles. Lycosidae, Araneidae, Anyphaenidae, Oecobiidae, and Thomisidae also had a high (> 50%) proportion of immature individuals (Table 1).

**Figure 2.**
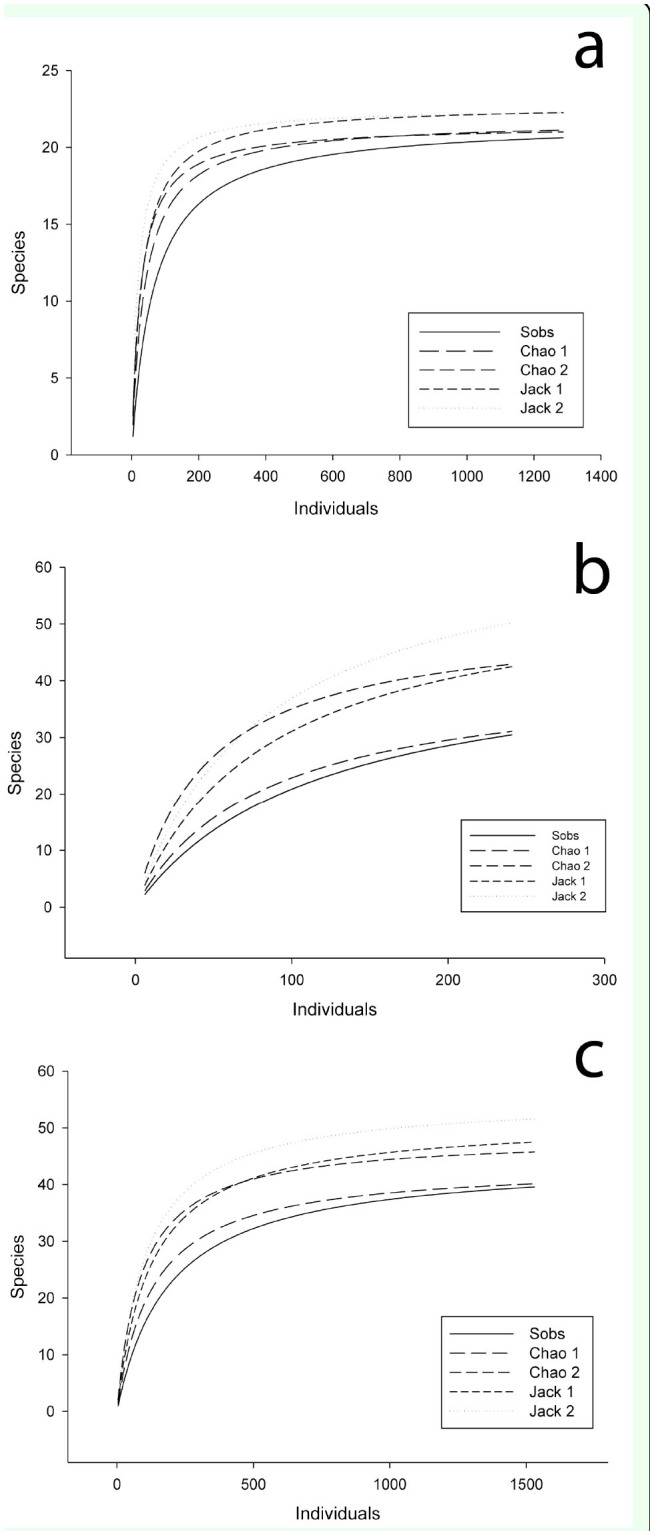
Estimates of spider richness in Fernando de Noronha according to sampling effort, considering: a) data from pitfall traps, b) data from active collection, and c) combined overall data. Calculations were performed using mean observed richness. Sobs: observed richness; Jack I : first-order jackknife; Jack 2: second-order jackknife. High quality figures are available online.

Of the 44 species, 42 (95.4%) had not been previously recorded for the island. Some specimens appeared to represent new species or belonged to species or genera not previously described from Brazil (Table 2). *Hogna* sp. (Lycosidae) was the most abundant species and represented 17.2% of total individuals collected. Several groups were recorded in low abundance, as 10 species were represented by a maximum of two individuals. Only five species were represented by more than 100 individuals, and their total abundance represented 64.5% of all adults collected (Table 2).

No species was found in all 10 sampled locations (Table 2), but *Carapoia* sp. nov., *Oonops* sp.l, and *Hogna* sp., in particular, were collected in the largest range of sites. Species richness varied according to the types of environment. The sites with the highest number of species were the preserved and urban areas, with 25 and 18 species respectively. The lodging and the preserved areas had the highest number of exclusive species, with six species each. Within-season species richness differed between the urban and preserved areas. In the dry season, 10 species in the urban area and only five in the preserved area were collected (F_(1,8)_ = 9.53; *p* = 0.015) (Table 3). In the rainy season, 11 species were caught in the preserved area and 14 in the urban area.

When only data from pitfall traps were considered, combining data from both seasons, the urban area presented a total of 15 species against 13 in the preserved area (Table 3).

Spider abundance in preserved vs. urban areas did not differ in the dry season, whereas in the rainy season the urban area had higher abundance than the preserved one, with 598 adults captured the former and 383 from the latter (Table 3). The indices of diversity and evenness were higher in the urban area (H' = 2.02; E= 0.76) compared to the preserved area (H' = 1.54; E = 0.62).

The results obtained by the use of species richness estimators varied from 41.86 to 55.95 species present on the island (Table 4). When the full data was used, the curves tend toward a plateau (Figure 2). However, when the full dataset is broken down into active and passive collecting methods, the latter showed a tendency to a plateau whereas the former tended to an ascendant curve.

Eight of the 11 guilds reported for Neotropical spider species were recorded (Table 5). A higher number of species was classified as diurnal space web-weavers, although, in terms of abundance, nocturnal ground hunters were dominant. When guilds were analyzed in relation to the type of environment, a greater range of behavioral and foraging strategies was detected in the preserved area (nine guilds) when compared to the urban area (six guilds).

## Discussion

### Overall spider diversity and guilds

This inventory revealed several new species, some of which have now been described (Ruiz et al. 2007; Brescovit et al. 2008; Rodrigues et al. 2008) or are under description. Prior to this study, only two species had been registered in Fernando de Noronha Island, *Cheiracanthium inclusum* (Bonaldo and Brescovit 1992) and *Umnara pydanieli* (Brescovit 1997). Since only juveniles of Miturgidae were captured, the total number of species on the island is higher.

The families Theridiidae and Salticidae stand out in terms of richness. Distributed world-wide, these are among the most specious families and exhibit potential for speciation through adaptative radiation in insular environments (Arnedo and Gillespie 2006). In general, the most abundant families in Fernando de Noronha are characterized by their ground-dwelling habits, a tendency partly due to the emphasis on soil collecting method and to the low diversity of vegetation in the island, which reduces the variety of microhabitat for web-spinning and other plant-associated species.

The substantial variation in abundance (from singletons to over 200 individuals) observed is a pattern similar to other tropical environments (Toti et al. 2000; Dias et al. 2005) in which a few dominant species contrast to many with low abundance. The high abundance of *Hogna* sp. can result from a positive selection effect by man-made devastation of reminiscent forests, as Lycosidae species are frequently encountered in open areas (Major et al. 2006).

The species estimation curves, when the data from active and passive collecting methods are separated, show that there is likely to be a large number of uncollected species, as the plateau of species accumulation curve is still to be reached. On the other hand, passive methods reached a plateau as if the collection effectiveness of this method had come to a maximum point of cost-benefit use.

The small size of the island and the unfavorable environment (no permanent freshwater source, low vegetation diversity) may act as limiting factors for the establishment of larger species. Low diversity and heterogeneity of species composition was documented in a field survey at the Atoll das Rocas (Almeida et al. 2000); in this unique atoll 266 km from the Brazilian coast that shares some characteristics with Fernando de Noronha islets, only four Araneae species were reported, one of them being *C. inclusum*, which was reported here.

Species classified in two guilds (nocturnal ground hunters and diurnal space web-weavers) corresponded to more than 70% of all individuals, although the description of spider guilds is an evolving classification. The low diversity and abundance of arboreal vegetation can account for the lower representation of orb weavers, since the availability of structural points of support and micro-habitats in the vegetation increases the diversity of orb weavers (Gollan et al. 2010). The leaf litter may facilitate the coexistence of several species, as the structural complexity of the litter increases the surface area and the diversity of foraging spaces within the leaves (Uetz 1991). In addition, the dry climate and intense exposure to sun radiation may favor nocturnal ground hunters and runners.

It is unclear whether these species also occupy the continental mainland or even other oceanic islands. Even if they are restricted to Fernando de Noronha, this proportion of endemism appears to be low when compared to other volcanic islands such as Hawaii (ca. 95%) (Eldredge and Miller 1995) and Galapagos (58%) (Baert et al. 1991), and this can be a direct effect of human activities related to the small size of the island.

### Influence of anthropogenic disturbance

The higher number of species found in urban areas, based on data obtained exclusively by pitfall traps, may result from the higher number of microhabitats available and the abundance of insect prey. Spider richness is affected by increased habitat heterogeneity, and the complex environment in urban areas promoted by human-mediated modifications included the introduction of almost 300 plant species (Teixeira et al. 2003). It is also relevant to point out that differences in conservation status are often linked to discrepancies in habitat heterogeneity, and that these features cannot always be dissociated from one another.

Vegetation structure influences spider diversity through several biotic and abiotic factors, such as structures for web support, temperature, humidity, shading, prey variety and abundance, refuges from natural enemies, and intraguild predation (Pinkus et al. 2006). The low vegetation diversity on the island provides limited microhabitats and fixation support for webs, relevant factors to spider communities (Greenstone 1984; Halaj et al. 1998). So, it is expected that the more complex urban environments that harbor exotic plant species are more diverse and are also likely to have a richer phytophagous community and a dense litter layer, two components that support the spider fauna (Uetz 1979; Halaj et al. 1998).

Complex mechanisms are suggested to explain the effect of habitat fragmentation on spider abundance (Bolger et al. 2000; Gibb and Hochuli 2002; Cardoso et al. 2010), including changes in prey availability, which can lead to local extinction in small fragments (Miyashita et al. 1998; Shochat et al. 2004). The higher richness and abundance values in the rainy season are not surprising, as periods with higher seasonal prey abundance are associated with more species and individuals of foraging predators.

From the 13 species present at the preserved site, only three were found in both seasons. In contrast, nine out of 15 species collected in the urban area were present in both dry and rainy seasons. This probably reflects a more changeable nature of the preserved area as a result of its vegetation characteristics (seasonal deciduous), which are likely to affect abiotic factors such as local temperature, humidity, sun exposure, and food availability.

Changes in environmental gradients brought about by human activities influence species distribution, altering the composition of communities (Uetz 1976). These changes can be evaluated by the combined use of Shannon's index and evenness values to compare community structures between sites. The lower values found in the preserved area may be due to the dominance of two species, *Coleosoma floridanum* Banks (Araneae: Theridiidae) and *Brignolia cubana* Dumitrescu and Georgescu (Oonopidae), which represented 96.6% of all specimens caught in the area. At the urban area, *Hogna* sp. and *Modisimus culicinus* (Simon) (Pholcidae) were the most abundant species, with 47% of total specimens, showing a less polarized distribution. So, despite the well-known negative effects of urbanization on arthropod diversity, some species actually benefit from this process in a short-term.

Data on the impact of urbanization on spider communities are controversial. The major response of spiders appears to be an overall decrease in diversity and an increase in total abundance when exposed to anthropogenic habitat alteration and fragmentation (Shochat et al. 2004), but this pattern was not detected here. In a long-term study in Arizona, moderate fragmentation of Sonoran desert into urban desert remnants did not reduce spider diversity; in fact, the number of species in desert fragments even exceeded that of desert conservation areas (Shochat et al. 2004). It may be that spider diversity peaks at intermediate levels of urbanization. Legal and logistical reasons impede that Fernando de Noronha from developing into a populous center, so a moderate degree of urbanization is unlikely to reduce total spider richness.

The short-term expeditions performed produce only a snapshot of local spider richness and could have underestimated the actual number of species. Spiders can migrate or disappear temporarily from their sites, and these events are important to the maintenance of species dynamics. However, the survey techniques employed were sufficient for mapping most of the diversity of soil-dwelling spiders, as the species accumulation curve indicates a plateau after the collection of 800 individuals. Extensive samplings that would allow for indepth analyses of seasonality effects were rendered impossible due to legal restraints on the numbers of individuals to be collected.

### Implications for conservation

The distance of Fernando de Noronha from the continent and its small size are limiting factors for a diverse endemic fauna to thrive. On the other hand, the history of exploration of its territory has been accompanied by intentional or accidental introductions of exotic species, including vertebrate predators and invertebrate competitors. Perhaps most dramatically, the massive destruction of local fauna and flora has reshaped the landscape in a degree that local extinction of native species is likely to have occurred. In a study performed at the Azores islands, Triantis et al. (2010) found out that forest-dependent spider species were among the most endangered taxa. When calculating a parameter known as “extinction debt,” they suggest that the severity of deforestation in the Azores (> 95%) has reduced dramatically the opportunities for forest species to cope with the increasing environmental changes.

Islands are well-known for elevated levels of arthropod extinction; for instance, the Hawaiian Islands have lost more arthropod species than the entire continental United States (Gillespie and Roderick 2002). One of the most severe impacts affecting arthropod populations on islands comes from non-native species, as extinctions attributed to alien species invasion have been documented (Miller and Eldredge 1996). Despite the protected status of Fernando de Noronha, domestic and international tourism grow rapidly, and this may be followed by the introduction of exotic invertebrate species (Freitas and Vasconcelos 2008). It is notable that from the five most abundant species, four are indisputably non-native (e.g., *Trachyzelotes kalczynskii* (Bösenberg) (Araneae: Gnaphosidae), *B. cubana).*

Island biotas display a relatively low dispersal capacity (reduced ability of ballooning, for example) so that under conditions of prolonged habitat modification, native species are likely to be replaced by alien species (Gillespie et al. 2008). So far, none of the species reported are classified as under risk of extinction in the International Union for Conservation of Nature red list of threatened species (IUCN 2010). Nevertheless, it can take several generations for the full impact of habitat destruction to produce extinction in oceanic islands (Tilman et al. 1994), so the true ecological cost on native biota is yet to emerge. The fact that the majority of documented extinctions since the 17^th^ century are species endemic to oceanic islands makes remote islands the priority areas for urgent conservation actions (Paulay 1994; Triantis et al. 2010).

From a conservation perspective, Fernando de Noronha can be compared to Easter Island, as both have suffered a complete alteration of the original environmental characteristics (Samways 2000). The composition of the island biota poses ecological and evolutionary challenges, such as the case of *Meioneta galapagosensis* Baert (Araneae: Linyphiidae), a species recorded previously only in the Galapagos and found on Fernando de Noronha without being reported in continental South America. Given the scant knowledge of arachnid diversity in northeastern Brazil, a direct comparison between communities in the archipelago and the adjoining continent is unattainable.

Several criteria should be met if spider species are to be considered priority for conservation, and a few of them apply to Fernando de Noronha. Martin et al. (2010) used weighed parameters such as ecological role, singularity, abundance, area of distribution, social value, threats, and potential for recovery, among others, to prioritize resource allocation for the conservation of oceanic island species. In Fernando de Noronha, restricted visits and awareness campaigns are directed chiefly to protect vertebrates, but arthropods are neglected. Inspection of cargo ships is insufficient to prevent the introduction of exotic species, and this risk has not yet been taken into account when tourism is expanded.

The high number of new and/or first recorded species on the island highlights the lack of studies on the invertebrate fauna of Southern Atlantic oceanic islands. Considering that data on the native and potential pool of non-native species is scarce, the identification of the indigenous spider fauna and the mechanisms for establishment, invasion, competition, and extinction remains a major challenge for conservation biologists in Fernando de Noronha. Since island colonization dynamics is a balance between extinction and immigration rates, the constant traffic of people and trade enhances the dispersion of alien organisms (Schoener and Spiller 1995), which can lead to an overall increase in invertebrate species richness, but the impacts of this on native species are unknown. The absence of systematic biodiversity surveys on the island continues to hinder management efforts for conservation.

**Table 1.**
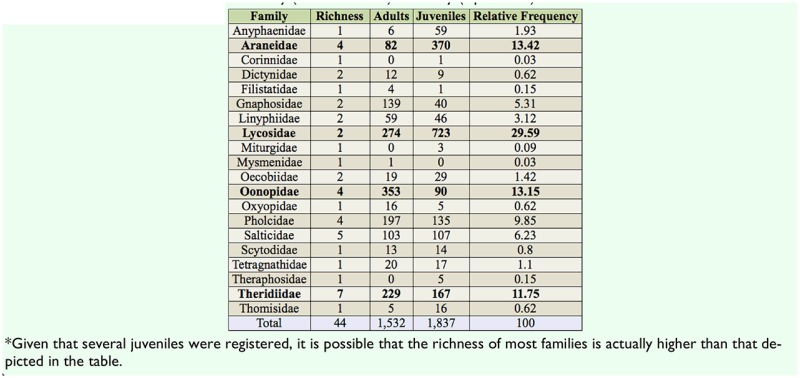
Species richness^*^ and abundance, according to developmental stage, of spider families collected on Fernando de Noronha Island combining data from the dry (October 2005) and rainy (April 2006) seasons. Families in bold are the most abundant (>10% of all individuals).

**Table 2.**
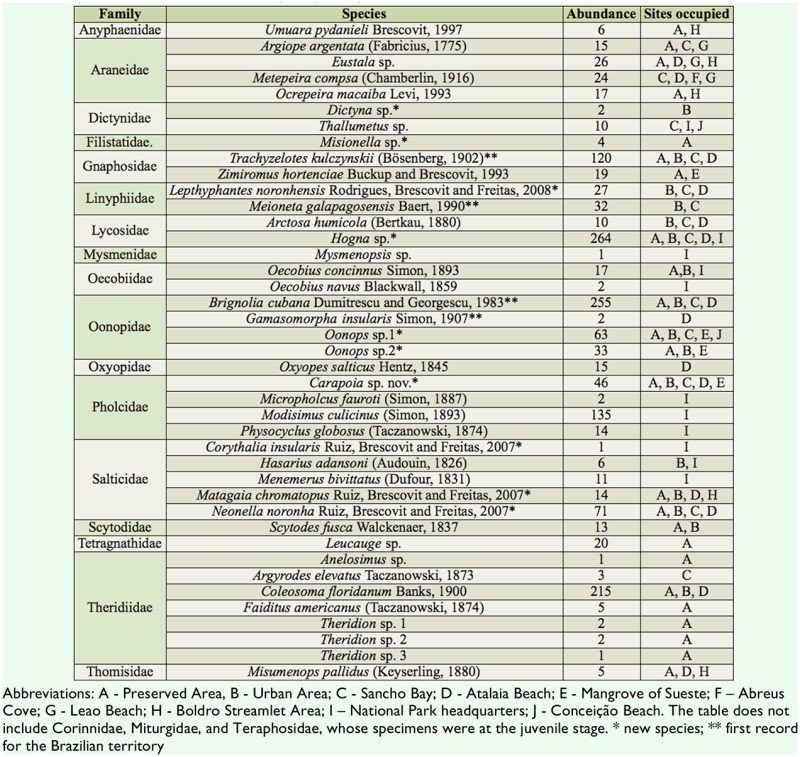
Spectrum, abundance, and locality of spider species collected on Fernando de Noronha Island.

**Table 3.**
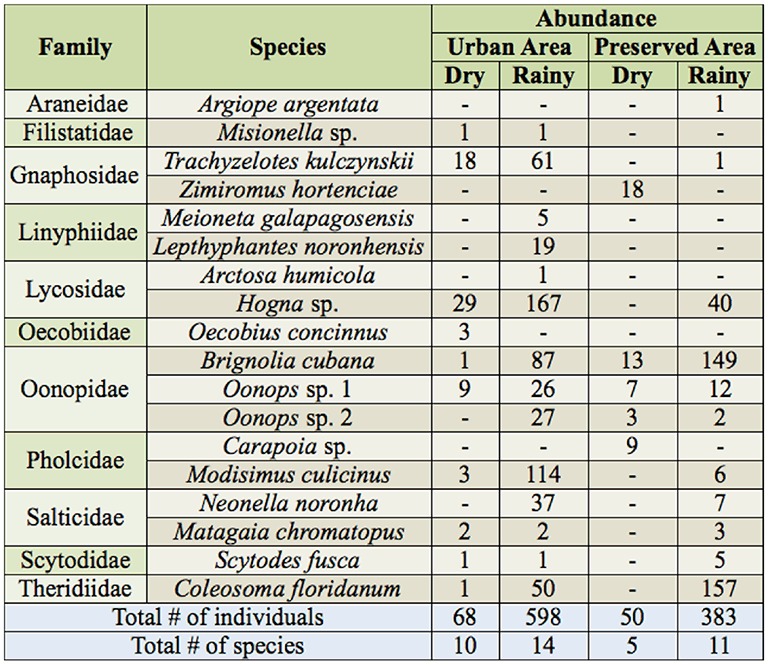
Comparison of the abundance of adult spider species collected with pitfall traps on Fernando de Noronha Island between preserved and disturbed areas and between seasons, according to collecting site and season.

**Table 4.**
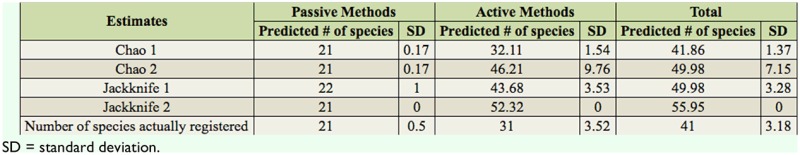
Comparison between sampling techniques of species richness of spiders collected on Fernando de Noronha Island as predicted by non-parametric species richness estimators.

**Table 5.**
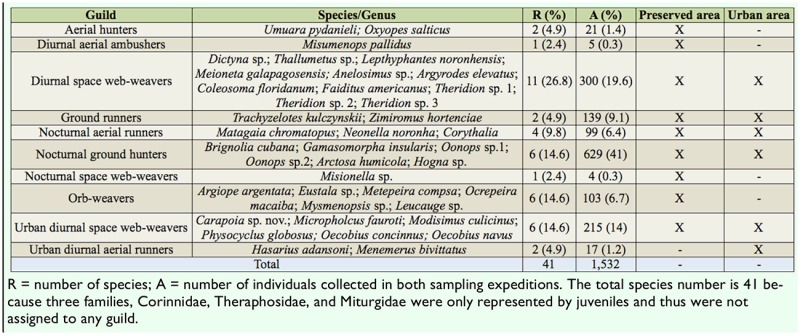
Classification of guilds of spider species present in Fernando de Noronha archipelago based on literature and the proposed reassessment of guilds in Neotropical spider species by Dias et al. (2010).
